# Making clinical decisions based on measurable residual disease improves the outcome in multiple myeloma

**DOI:** 10.1186/s13045-021-01135-w

**Published:** 2021-08-17

**Authors:** Joaquin Martinez-Lopez, Rafael Alonso, Sandy W. Wong, Rafael Rios, Nina Shah, Yanira Ruiz-Heredia, Jose Maria Sanchez-Pina, Ricardo Sanchez, Natasha Bahri, Irene Zamanillo, Maria Poza, Natalia Buenache, Cristina Encinas, Luis Juarez, Fatima Miras, Luis Collado, Santiago Barrio, Thomas Martin, Maria Teresa Cedena, Jeffrey Wolf

**Affiliations:** 1 Hematology Department, Hospital 12 de Octubre i+12, CNIO, Complutense University, Madrid, Spain; 2 Department of Medicine, Division of Hematology-Oncology, University of California San Francisco, San Francisco, USA; 3 Hematology Department, Hospital Virgen de Las Nieves, Granada, Spain; 4 Hematology Department, Hospital General Univesitario Gregorio Marañon, Madrid, Spain; 5 Medicine Department, Complutense University, Madrid, Spain

**Keywords:** Measurable residual disease, Multiple myeloma, Minimal residual disease

## Abstract

The assessment of measurable residual disease (MRD) in bone marrow has proven of prognostic relevance in patients with multiple myeloma (MM). Nevertheless, and unlike other hematologic malignancies, the use of MRD results to make clinical decisions in MM has been underexplored to date. In this retrospective study, we present the results from a multinational and multicenter series of 400 patients with MRD monitoring during front-line therapy with the aim of exploring how clinical decisions made based on those MRD results affected outcomes. As expected, achievement of MRD negativity at any point was associated with improved PFS versus persistent MRD positivity (median PFS 104 vs. 45 months, *p* < 0.0001). In addition, however, 67 out of 400 patients underwent a clinical decision (treatment discontinuation, intensification or initiation of a new therapy) based on MRD results. Those patients in whom a treatment change was made showed a prolonged PFS in comparison with those 333 patients in which MRD results were not acted upon (respectively, mPFS 104 vs. 62 months, *p* = 0.005). In patients who achieved MRD negativity during maintenance (*n* = 186) on at least one occasion, stopping therapy in 24 patients vs. continuing in 162 did not alter PFS (mPFS 120 months vs. 82 months, *p* = 0.1). Most importantly, however, in patients with a positive MRD during maintenance (*n* = 214), a clinical decision (either intensification or change of therapy) (*n* = 43) resulted in better PFS compared to patients in whom no adjustment was made (*n* = 171) (mPFS NA vs. 39 months, *p* = 0.02). Interestingly, there were no significant differences when MRD was assessed by flow cytometry or by next-generation sequencing. Herein, we find that MRD is useful in guiding clinical decisions during initial therapy and has a positive impact on PFS in MM patients. This potentially opens a new dimension for the use of MRD in MM, but this role still remains to be confirmed in prospective, randomized clinical trials.

To the Editor,

The assessment of bone marrow measurable residual disease (MRD) has consistently shown a significant prognostic value in patients with multiple myeloma (MM), with a benefit in survival outcomes associated with MRD negativity surpassing the value of complete response [1, 2]. Next-generation sequencing and Euroflow on bone marrow reach higher sensitivity than standard flow, increasing the predictive potential [3, 4]. Thus, MRD was included in the consensus criteria for response [5] and its role as a surrogate marker for survival outcomes is under consideration [1, 6]. Preliminary studies suggest that MRD dynamics could demonstrate greater prognostic value than just the MRD status at a single time point [7, 8].

MRD assessments are performed in MM to assess the quality of response and to make prognostic statements, but one can imagine using such results to make clinical decisions, much as one does with M-spikes. Unlike other hematological malignancies, therapeutic decisions (treatment escalation, de-escalation or discontinuation) based on MRD is a pending topic in MM.

Korde et al. [9] published a trial where MRD testing impacted the number of cycles of therapy as it is also planned in the MASTER trial [10]. Following IFM2009 trial, some have postulated the usefulness of post-induction MRD status to decide between early/delayed autologous stem cell transplantation [11]. Also, ongoing trials as REMNANT or PREDATOR-MRD are evaluating the role of MRD conversion (from negative-to-positive) as a trigger for pre-emptive therapy [12]. However, these results are still preliminary and conclusive data are scarce.

We analyzed how MRD results could guide clinical decision-making through the retrospective analysis of outcomes in 400 MM patients with extensive MRD monitoring during frontline therapy (patients at least in VGPR and ≥ 1 MRD assessments during follow-up according to our clinical practice). NGS of Ig genes or second-generation flow or next generation flow at level of 10^−5^ was employed for MRD assessment and 92% of patients were in CR. In 67 patients, a clinical decision was made based on MRD results, mostly during maintenance (83%). Thirty-three out of these 67 were MRD-negative cases (treatment was reduced in 3 and stopped in 30), while 34 were MRD-positive when a therapy change was made (intensification in 27 and new treatment in 7 cases). None of them met criteria for progressive disease according to IMWG consensus. Twelve out of 34 MRD-positive patients subsequently achieved MRD negativity after intensification or a change in therapy.

Globally, 186 patients achieved MRD negativity showing a prolonged progression-free survival (PFS) versus those who did not achieve MRD negativity (mPFS 104 vs. 45 months, *p* < 0.0001). No differences were observed when MRD was assessed by NGS or MFC (*p* = 0.2).

Patients in whom a clinical decision was made based on MRD (*n* = 67) had a prolonged PFS versus those in whom a clinical decision was not made (*n* = 333) (mPFS from the first MRD datapoint was 104 months [73–165] vs. 62 months [46–80], *p* = 0.005); statistical significance persisted in a landmark analysis at 12 months (*p* = 0.04) or from the start of induction (*p* = 0.05) (Fig. 1a). No differences in major clinical features were found between both subgroups (Table 1a–d). In the MRD-negative group, those in whom treatment was stopped did just as well as those whose therapy was continued (mPFS, 120 vs. 82 months, *p* = 0.1). Patients with an MRD-positive marrow, in whom therapy was changed or intensified, exhibited prolonged PFS versus those who continued therapy without change (mPFS, Not reached vs. 39 months, *p* = 0.02) (Fig. 1b, c). Only making clinical decisions based on MRD (HR 0.5; 95%CI 1.41–6.87) and age (HR 1.2; 95%CI 1.1–1.5) were significant in a multivariate analysis (including age, sex, myeloma isotype, cytogenetic risk, hemoglobin, response, creatinine and clinical decision-making).

**Fig. 1:**
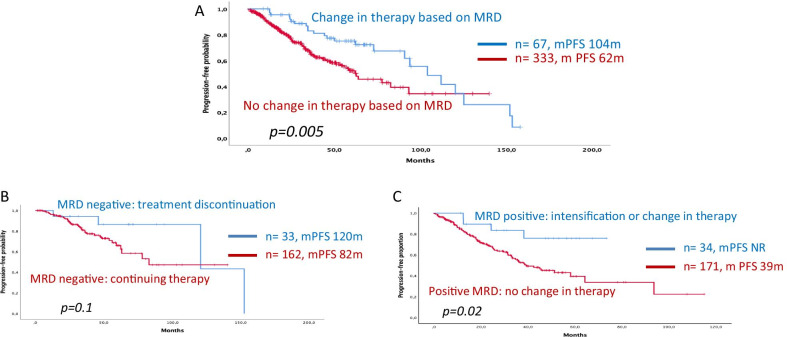
Kaplan–Meier curves showing the impact of making clinical decisions based on MRD. **a** PFS from the first MRD datapoint, comparing patients who underwent a change in therapy based on MRD with those in whom no change in therapy was made. **b** MRD-negative patients: treatment discontinuation (maintenance or transplant) vs. no change in therapy. **c** MRD-positive patients: beginning a new therapy or intensifying therapy vs. no change in therapy

**Table 1:** Main characteristics at diagnosis and therapy at frontline, all patients (*n* = 400)

	Overall (*n* = 400)	No change in therapy based on MRD (*n* = 333)	Change in therapy based on MRD (*n* = 67)
Male (%)	55	57	45
Mean age, years (sd)	58.9 (0.4)	59.2 (0.7)	57.1 (1.3)
Myeloma type, (%)			
IgG	55	56	46
IgA	19	19	20
Light chain only	19	17	27
High risk cytogenetics*, (%)	17	17	16
ISS, (%)			
I	38	35	40
II	26	28	38
III	35	33	27
Hemoglobin, (g/dL) (sd)	12.5 (0.7)	12.6 (0.7)	12.1 (1.2)
High LDH levels, (%)	20	20	21
Induction treatment, (%)			
CyBorD	19	19	19
VRD/VTD	45	49	40
Others	36	32	41
ASCT, yes (%)	78	78	81
Maintenance, yes (%)	82	82	91

CyBorD: bortezomib, cyclophosphamide, dexamethasone; VRD: bortezomib, lenalidomide, dexamethasone; VTD: bortezomib, thalidomide, dexamethasone; ASCT: Autologous Stem Cell Transplantation. When we compared both groups, we did not find any statistical significance, *p* > 0.05 in all comparisons

^*^High-risk cytogenetics was defined as del 17p; *t*(4;14), *t*(14;16) or *t*(14;20). When we compared both groups, we did not find any statistical significance, *p* > 0.05 in all comparisons

Depth of MRD is commonly considered the best prognostic factor in MM [2, 4, 7], and a good surrogate marker for survival in clinical trials [1]. However, some myeloma experts question the employment of MRD to guide MM treatment due to the lack of evidence. Our results suggest that the use of MRD to make clinical decisions has a positive impact on survival outcomes.

The achievement of MRD negativity had a relevant impact on PFS. Interestingly, PFS improved when treatment was modified in patients who were MRD-positive; while PFS was not different according to the discontinuation or persistence of therapy when MRD negativity was achieved. The main limitations of this study are its retrospective nature, the heterogeneity of the time of MRD assessment, and the lack of specific pre-defined rules regarding when and how to make these decisions and the small sample size of the MRD making decisions population, for these reasons, results should be interpreted carefully. Nevertheless, this study has several strengths including the large sample size and the multinational and multi-institutional approach with superimposable results between methodologies and institutions.

In conclusion, the use of MRD to guide treatment in MM is potentially as useful as Serum Protein Electrophoresis and light chain measurement, especially in patients who are in stringent Complete Response. Prospective randomized clinical trials currently ongoing may provide new evidence in this setting.

## Data Availability

The datasets used and/or analyzed during the current study are available from the corresponding author on reasonable request.

## Abbreviations

MRD: Measurable residual disease
MM: Multiple myeloma
VGPR: Very good partial response
NGS: Next generation sequencing
PFS: Progression free survival
mPFS: median Progression free survival
